# A Case-Study of Metoclopramide Prescription Error : A Grim Reminder

**DOI:** 10.1007/s10916-024-02099-3

**Published:** 2024-08-22

**Authors:** Florent Wallet, Charlotte Doudet, Alexandre Theissen, Arnaud Friggeri, Charles-Hervé Vacheron

**Affiliations:** 1 PHE3ID, Centre International de Recherche en Infectiologie, Service d’Anesthésie Réanimation – Médecine Intensive, Institut National de la Santé et de la Recherche Médicale U1111, CNRS Unité Mixte de Recherche 5308, École Nationale Supérieure de Lyon, Université Claude Bernard Lyon 1, Centre Hospitalier Lyon Sud, Hospices Civils de Lyon, Lyon, France; 2 Service de pharmacie, Centre Hospitalier Lyon Sud, Hospices Civils de Lyon, Lyon, France; 3 Anesthésie - Réanimation Chirurgicale Clinique Saint François, Pierre-Bénite, France; 4 Département d’Anesthésie Réanimation, Centre Hospitalier Lyon Sud Hospices Civils de Lyon, Pierre-Bénite, France; 5 Faculté de Médecine Lyon Est, CIRI, Centre International de Recherche en Infectiologie (Equipe Laboratoire des Pathogènes Emergents), Inserm, U1111, Université Claude Bernard Lyon 1, CNRS, UMR5308, Lyon, France; 6 Service de Biostatistique - Bioinformatique, Hospices Civils de Lyon, Pôle Santé Publique, Lyon, France; 7 Centre Hospitalier Lyon Sud Hospices Civils de Lyon, Service d’anesthésie- réanimation, 165 chemin du Grand Revoyet, Pierre-Benite, 69495 France

**Keywords:** Medication error, Patient safety, Nurse workload

## Abstract

The integration of Computerized Provider Order Entry (CPOE) systems in hospitals has been instrumental in reducing medication errors and enhancing patient safety. This study examines the implications of a software oversight in a CPOE system : Metoclopramide had a concentrated formulation (100 mg) delisted (and then not manufactured) in 2014 due to safety concerns. Despite this, the CPOE system continued to accept prescriptions for this formulation because it was not removed from the medication library by the pharmacist. The objective of our study was to describe this specific prescription error related to an outdated the medication library of the CPOE. We analyzed all metoclopramide prescriptions from 2014, to 2023. Our findings showed that errors involving 100 mg or more dosages were relatively rare, at 2.98 per 1000 prescriptions (34 errors in 11,372 prescriptions). Notably, 47.1% of these errors occurred during on-call shifts, and 68% of these errors led to actual administration. These errors correlated with periods of higher nurse workload. The findings advocate for the integration of dedicated pharmacists into ICU teams to minimize medication errors and enhance patient outcomes, and a proactive medication management in healthcare.

## Brief Technical Report

The integration of Computerized Provider Order Entry (CPOE) systems in hospitals represents a significant step towards enhancing patient safety. Their adoption has been synonymous with a marked reduction in medication errors. However, it is worth noting that with the implementation of CPOE, we are also witnessing the emergence of new, unique types of errors [1]. These errors, though present, have their incidence and implications not fully documented in current medical literature. Metoclopramide, a drug primarily prescribed for the prevention and treatment of nausea and vomiting, is frequently used in intensive care settings. Conventionally, it is available in 10 mg ampoules, with dosage recommendations spanning from 10 to 30 mg per day. In France, until 2014, a more concentrated formulation of metoclopramide (100 mg ampoules) was available. This formulation was used for managing nausea and vomiting in patients undergoing chemotherapy or radiotherapy and was not used in the ICU setting. It was, however, delisted (and therefore not manufactured) from the French market in 2014 owing to concerns about its benefit-risk profile [2].

Remarkably, a software oversight in our CPOE (IntelliSpace Critical Care and Anesthesia^®^, Phillips, Amsterdam) dedicated to our ICU, and used for the physician prescription still accepted prescriptions for the 100 mg metoclopramide formulation. This loophole was due to a lack of update of the medication library of the CPOE by the pharmacist, left room for errors, particularly if the prescribing physician was not adequately vigilant : indeed, a physician who wishes to prescribe ‘one ampoule’ of metoclopramide could choose an ampoule containing 100 mg of metoclopramide. Therefore this led to a prescription error were the physician could prescribe one or two ampoule of metoclopramide 100 mg (i.e. 100 or 200 mg). On the 29/08/2023, this error was detected by a physician and corrected in the medication library of our CPOE by the pharmacist.

We therefore reviewed all metoclopramide prescriptions from 12/02/2014 to 29/08/2023 in a French university Intensive Care Unit (ICU). No exclusion criteria were applied. Our findings revealed that prescribing errors, were relatively infrequent, accounting for 2.98 per 1000 prescriptions (34 errors from a total of 11,372 metoclopramide prescriptions). A closer look revealed that 47.1% of these errors took place during on-call shifts (between 6pm to 8 am). Additionally, 68% (*n* = 23) of these prescription errors led to an administration of metoclopramide (informatic confirmation of administration). It was evident from our study that errors coincided with periods when nurses faced a higher workload, especially compared to standard on-call periods, since the validation of the metoclopramide was associated with a higher NEMS (Nine Equivalents of nursing Manpower use Score) for the patient (mean NEMS 6.75 ± 3.87 vs. 12.5 ± 7.13, p value of the Wilcoxon test < 0.001). Briefly, the NEMS is a well validated tool to estimate the nurse workload per ICU patient based on care required, ranging from 0 (low workload) to 66 (high workload) points [3].

No adverse events (such as extrapyramidal syndrome or cardiovascular disorders) were reported in relation to metoclopramide inappropriately high dosage. This can be explained by the nurse correcting the dosage during the administration, or due to metoclopramide broad therapeutic window [4]. However, this serves as a grim reminder of the potential consequences that could arise from similar oversights with high-risk medications. This is especially true for drugs like catecholamines or sedatives, where the margin for error is minimal and the stakes are significantly higher. The same caution applies to drugs with a wider therapeutic margin but with the potential for severe adverse drug events, such as insulin or opioids.

An important aspect of this study that warrants further discussion is the direct correlation between the workload of nursing staff and the incidence of medication errors. Our findings notably revealed that a substantial proportion of these errors occurred during on-call shifts, a time typically characterized by increased workload and reduced staffing levels. This correlation is more than coincidental; it highlights a systemic issue in healthcare settings where the workload and staffing ratios significantly impact the likelihood of errors. The complexity of these tasks, combined with a high patient-to-nurse ratio, can lead to a heightened risk of errors. This situation is exacerbated during on-call hours when fatigue and reduced vigilance further impair their ability to catch and correct potential errors [5].

Most importantly, this observation confirms the growing evidence that pharmacists are a crucial part of the ICU team, emphasizing the need for a dedicated pharmacist in each ICU unit [6]. The role of a pharmacist in the ICU is specialized, contributing significantly to patient safety, and it requires specific qualifications for ICU pharmacovigilance [7, 8]. In this instance, a pharmacist dedicated to this specific ICU’ would have update the medication library by removing the incorrect metoclopramide formulation in 2014. Alternatively, by reviewing the medications of patients admitted to the ICU, this error could have been avoided and likely would not have persisted for nearly 10 years.

In conclusion, while CPOE systems have been instrumental in enhancing patient safety, our study reveals that technological solutions alone are not sufficient. There is a pressing need for a holistic approach that addresses the human factors in healthcare, such as nursing workload and interdisciplinary collaboration. By recognizing and addressing these factors, we can significantly reduce the incidence of medication errors and, consequently, improve the overall quality of patient care in ICU settings [9–11]. Furthermore, an ICU pharmacist managing these CPOE systems would optimize interventions to prevent prescription errors. Such interventions could include restricting prescriptions, issuing alerts about incorrect dosing or drug interactions, and enhancing the overall appropriateness of medication use [12, 13].

This study thus sheds light on the need for more robust workload management strategies in healthcare settings. Adequately staffing shifts, particularly during high-risk periods like nights and weekends, and ensuring reasonable patient-to-nurse ratios are essential steps in mitigating medication errors. Additionally, providing support systems such as regular breaks, adequate rest periods, and mental health resources can significantly reduce fatigue-related errors.

## Data Availability

No datasets were generated or analysed during the current study.
